# Ligaturing One of the Common Corotid Arteries, for the Cure of Epilepsy

**Published:** 1857-10

**Authors:** C. Angell

**Affiliations:** Pittsburgh, Indiana


ARTICLE III.—*Ligaturing one of the common Corotid Arteries, for the cure of Epilepsy.* By C. ANGELL, of Pittsburgh, Indiana.

Have we any known remedy for the cure of Epilepsy? Without attempting to explain the nature, cause or phenomena of this disease, I will present the result of two operations of tying the right common Corotid, for the cure of this disease.

The first operation was performed on the 2d day of July, 1857, on Wm. Brackus, of this place. He was 20 years of age, short, not over 5 feet in hight, heavy set, large head, short neck, full habit, sanguine temperament, right arm and hand deformed, idiotic in most respects. Has been having fits 3 or 4 years, seldom at first, but gradually growing in frequency and severity. After having had a variety of remedies tried without any good result, I proposed tying one of the common Corotids, thinking that by permanently lessening the amount of blood passing to the brain that it might cure him. After having had 15 or 20 in the fore part of the day, being put under the influence of Chloroform, I placed a ligature on the right common

Corotid, about one inch below the bifurcation of the external and internal. The operation was performed without any difficulty.

The next day pulse 120, complains of difficulty in swallowing, talks with difficulty, complains of no pain, his mind about as it was before the operation, left side partially paralyzed.

Treatment: solution of Salts and Tar. Emetic, as a cathartic and sedative. They operate well, wound covered with oil silk, and kept constantly wet with ice water; gave nothing to eat but water gruel and rice water.

Without describing each particular day, I will say that he continued about as I have described him to-day, paralysis of the left arm and leg complete, still talks and swallows with difficulty, not much swelling about the wound and neck.

He remained in this condition until the night of the seventh day from the operation, when he died in a comatose condition. He never had any symptoms of fits after the operation.

An examination of the neck the next morning after he died, showed no signs of inflammation, the parts all looked healthy and natural. I removed a section of the artery two inches in length, above and below the ligature, coagulation was quite firm in artery. The family think he would have died as soon as he did, if he had not been operated upon. I do not think so.

The second operation was performed on the 8th of July, the same day on which the first one died. This was on J. Bostick, of Brookston. He is 40 years of age, naturally good constitution, full habit, sanguine temperament. Has had fits for seven years, but not so often or so hard, until the last three years; has them almost every day, so much so that he is incapacitated from doing any thing at all, his mind is also destroyed. He has been under treatment by a great many, both regular and irregular, all without benefiting him.

I performed the operation in the same way as in the other case. Soon came out from under the influence of the Chloroform, said he had complained of no pain, talked without any difficulty.

July 8th.—Says he feels well to-day, pulse 80, no unfavorable symptoms, swallows well, mind clear, talks well. I bled him this morning very freely, and put him on tar. emt. and salts, as in the other case. He has continued to get along well, pulse never over 80. He would every few days wake up bewildered or scared, this being the only symptom of returning fits until the 30th. He had one on this day so hard as to deprive him of sensibility, and on the next day had one about the same. He says they came on him different from what they did before. Before this he would have no warning or premonitory symptoms of their approach; but each one of these times he felt a sensation of dizziness of some minutes' duration, long enough for him to go and lay down before they came on.

On the 17th of August he had a slight one, and on the 5th of September another, making in all four since the operation: it is now the 18th of September.

The ligature came away on the twenty-second day—the wound is all healed up. He is going about attending to his business. He says he feels better than he has done in three years.

His family, and those that are well acquainted with him, all say that they can see a marked change in him, in his actions, countenance, and more particularly in his mind.

What will be the result I cannot tell. He now has the appearance of being benefited; how long it will continue I cannot determine. Whether the bettering of his condition is owing to the operation, or to the treatment, I cannot say.

Any remarks on the above cases will be thankfully received.

